# Life history strategy and overeating during COVID-19 pandemic: a moderated mediation model of sense of control and coronavirus stress

**DOI:** 10.1186/s40337-021-00514-5

**Published:** 2021-12-09

**Authors:** Baojuan Ye, Ruining Wang, Mingfan Liu, Xinqiang Wang, Qiang Yang

**Affiliations:** 1 Center of Mental Health Education and Research, School of Psychology, Jiangxi Normal University, 99 Ziyang Avenue, Nanchang, 330022 China; 2 School of Education, Jiangxi Normal University, 99 Ziyang Avenue, Nanchang, 330022 China

**Keywords:** Life history strategy, Sense of control, Coronavirus stress, Overeating, College students, COVID-19

## Abstract

**Background:**

This study examined the mediating effect of sense of control and the moderating effect of coronavirus stress on the relationship between life history strategy and overeating among Chinese college students during the COVID-19 period.

**Methods:**

3310 Chinese students (*M*_*age*_ = 19.74, *SD* = 1.50; 47% males) completed self-reported questionnaires regarding life history strategy, sense of control, overeating, and coronavirus stress. The data were analyzed using Pearson’s r correlations and moderated mediation analysis.

**Results:**

The results revealed that control sense mediated the link between life history strategy and college students’ overeating. College students’ coronavirus stress moderated the associations between life history strategy and college students’ sense of control and between control sense and overeating. The association between life history strategy and sense of control was stronger for those with lower coronavirus stress, and the association between sense of control and overeating was stronger for those with lower coronavirus stress.

**Conclusions:**

This study identified that the critical factors were associated with overeating. On the one hand, the research deepens the application and interpretation of life history theory in the field of eating; on the other hand, it provides evidence for the prevention of overeating, and provides theoretical basis for psychological assistance among Chinese college students.

## Plain English summary

One’s response is not arbitrary when confronted with COVID-19 pandemic. Previous research has found that individuals with fast life history strategies still be more prone to overeat. We also know from other research that sense of control is the most salient aspect of overeating. However, no research has investigated whether the life history strategy of college students is significantly associated with overeating and examine the potential mediating and moderating mechanisms in this association. With the aid of 3310 participants, we found that, life history strategy was linked to college students’ overeating. We also found that this relationship was partially explained by sense of control. In addition, the association between life history strategy and sense of control was stronger for those with lower coronavirus stress, and the association between sense of control and overeating was stronger for those with lower coronavirus stress. This study is an important step in unpacking how life history strategy relates to overeating in Chinese college students. However, they are limited by the cross-sectional nature of the study, meaning we cannot imply causality. We recommend that further research replicate our findings in people with diagnosed eating disorders using a longitudinal design.

## Introduction

The novel coronavirus disease (COVID-19) is an infectious respiratory virus, declared a global pandemic on 11th March 2020 [1]. The global pandemic of COVID-19 has caused radical changes in the structure of people’s daily routines in most of the countries around the world, including people`s food intake [2–4]. Previous studies have suggested an increase in anxiety and depressive symptoms which negative impacts on psychological health could provoke the overeating of unhealthy food and result in weight gain [5–9]. Recent research reported that many individuals report increased binge eating, overeating, using food to cope with Coronavirus stress, and unhealthy food intake during the COVID-19 pandemic [10–12]. Previous study has determined that university students lack adequate and balanced meals due to their separation from their families, insufficient funds, or lack of time and information about healthy nutrition [13].

Previous research has found that overeating is a relative term. Short-term overeating is a common human habit associated with feasting and celebration. Overeating becomes a health risk when is sustained over long periods. The fundamentals of the energy balance equation dictate that long-term overeating will always lead to body fat storage and obesity [14]. Overeating defined as eating of large amounts of food irrespective of loss of control when eating [15] Overeating is not only a direct cause of obesity and eating disorders, but also an important risk factor for internalization and externalization problems such as depression, anxiety, self-harm, and substance abuse [16]. As we all know, diet is a key driver of health [17] and well-being [18, 19], not only through the provision of nutrients [20] or patterns of consumption [17], but also the social health benefits of shared meals [21]. In other countries, the COVID-19 pandemic and associated containment strategies are thought to have affect eating behaviour [22–24]. In Italy, one survey found that around 53% of respondents reported eating more during lockdown and 19.5% reported weight gain [25]. Another survey of adults in Poland, more than 40% respondents reported eating more and 42% respondents reported weight change (loss or gain) [26]. Aside from the society-wide impact of confinement on diet, loss of the sense of taste and smell is a common side effect in those who infected with COVID-19 [27], which may also affect dietary intake.

From a psychosocial perspective, the outbreak of COVID-19 can be considered as an acute stressful or traumatic event which can lead to the emergence of various sorts of psychological problems [28–30]. Individuals is different in their reaction propensity to experience negative life events. When confronted with challenge or environmental uncertainty (e. g. COVID-19 pandemic), one’s response is not arbitrary. In the recent literature, evolutionary-informed theories aimed to explain such individual differences have become more prevalent [31–33]. One of those theories is the Life History theory (LHT), which was initially focused on explaining differences in reproductive strategies between species [34]. At the core of life history theory is the appreciation for the enduring influence of information in early development being utilized as a forecast in service of meeting the environmental demands of later development [35, 36]. Life history strategies exist along a “slow” to “fast” continuum – terms that indicate the relative tempo of one’s development and reproduction [35]. The fast strategy and the slow strategy constitute the two poles of the life history strategy continuum. The weighing result of the individual determines its position in the continuum, and leads to the formation of corresponding personality traits as the executive body, coordinating behavior and the environment to work together to complete the adaptation task [37]. Slow strategists are characterized by stable relationships (kin, romantic, social exchange partners) and a propensity for long term planning, risk averseness, and prosocial behavior [38]. Fast life histories are marked by the opposite pattern. Fast strategists accelerate development and develop an orientation toward succeeding in the here and now, so they are more inclined to enjoy the current pursuit of instant gratification which include risk-taking, short-term decision making, and reduce prosocial behaviors [38–40]. In other words, when individuals are facing the negative effect of the COVID-19 pandemic, individuals with a slow life history strategy will solve the problem in a more constructive way, while individuals with a fast life history strategy will do the opposite. Although food scarcity has been improved in the modern world, individuals with fast life history strategies may still be more prone to overeat, because fast life history strategies are associated with high levels of impulsivity and instant gratification, and delicious foods can satisfy an individual’s need for instant gratification [41]. In addition, both cross-sectional and longitudinal studies found a positive association between fast life history strategies and addictive behavior such as smoking and alcohol abuse [42, 43], as well as problematic eating behaviors [44, 45]. Salmon et al. [45] found that the fast life history strategy significantly predicted increased eating disorder symptoms. In other words, individuals with fast life history strategies during the COVID-19 pandemic may be more inclined to overeating.

Given this theoretical framework, the aim of the present study is to investigate whether the life history strategy of college students is significantly associated with overeating and examine the potential mediating and moderating mechanisms in this association.

### Sense of control as a mediator

It is important to consider the possible mediators that may play a role in decreasing the levels of overeating. In particular, sense of control may be a subsequent consequence of different life history strategy as well as being an antecedent of overeating. Sense of control is a central construct in psychology, and describes a basic motivational variable shaping one’s adaption to life and coping with life stress. As Bandura [46] observed, “among the mechanisms of personal agency, none is more central or pervasive than people’s beliefs in their capacity to exercise some measures to control their own functioning and environmental events” (p. 10). Sense of control is a psychological driving factor for behaviors related to life history strategies [47]. Previous study shows that sense of control is significantly related to life history strategies [48]. There are many studies that regard control as a component of life history strategy [33, 49], but from a more rigorous point of view, the fluctuating changes and levels of the sense of control are the result of internal trade-offs in the individual's life history strategies. According to the internal mechanism of the life history strategy which means that individual’s early experience influences the formation of life history strategies, which in turn influences current behavior (i.e., sense of control) [50–53]. Wang et al. [54] point out that life history strategy is a choice of behavioral strategy driven by intrinsic motivation, and this motivation is to obtain and maintain sense of control. Empirical research shows that the life history strategy is a stable behavioral strategy choice for lone-term life which affect changes in individuals’ sense of control [54, 55]. Fast strategists often maintain individuals’ sense of control through irrational behaviors such as current squandering and instant gratification [56], while slow strategists tend to maintain and improve individuals’ sense of control by reducing risky behaviors [57].

While past studies have evidenced that sense of control predicts a diverse range of overeating, no studies to date have directly investigated the mediating role of sense of control in the association between life history strategy and overeating in college students. While past studies have evidenced that sense of control is the most salient aspect of overeating [58], and that this feature is more strongly associated with psychiatric symptomatology than the quantity of food consumed [59]. In the fields of obesity and eating disorders, studies have found that low control ability can cause people to experience overeating symptoms [60]. Perceived control is associated with eating disorders recovery during the COVID-19 pandemic, with higher perceptions of external control being associated with recovery [61]. The higher the level of individual control, the higher the level of regulation of their overeating behavior [62–64].

Given the uncertainty associated with the course of COVID-19, it stands to reason those students with low life history strategy may perceived more sense of control, which in turn may decrease their risk for overeating.

### Coronavirus stress as a moderator

Although life history strategy may impact college students’ level of overeating through the mediating role of sense of control, and individuals with different life history strategies respond differently to stressors, not all college students with low life history strategy may perceive a high level of sense of control. One key buffering mechanism may be coronavirus stress.

COVID-19 is a health threat identified as a significant stressor threatening the mental health and well-being of many individuals around the world [65–67]. It has been suggested that COVID-19 stress can trigger mild to severe levels of psychosocial problems, such as depression, somatization, and anxiety [65, 67–69]. There is extensive evidence that stress undermines the individual’s sense of control [70–72]. In Malinowski's research, for fishermen who feel higher stress, they have lower sense of control over life events [73, 74]. This is because stress coping involves the control of attention, thinking, and emotions, and this process itself is a process of consuming sense of control [75–77]. Based on past researches, uncertainties triggered by the COVID-19 crisis might bring about adverse outcomes especially for individuals who have a low sense of control, including an increase in the likelihood of these individuals’ unhealthy behaviors [78] (e.g., overeating). Thus, as stress increases, the negative effect of fast life history strategies is diminished and sense of control increases.

In addition, the psychological impact of stress is reflected in the fact that stress can lead to changes in the individual’s cognition, emotions, and coping styles, thereby affecting food choices. These changes can occur alone or interact [79]. Born et al. [80] has found that subjects who experienced more stress pursued a richer taste experience than those in the control group who experienced less stress, which is in line with the view of escape theory. Central to escape theory is the notion of multiple levels of meaning, which are linked to multiple ways of being aware of oneself and one's activities [81–83]. The escape model provides a frame work for viewing how individuals might escape the aversive negative affective state [84–86]. In this view, the reason why an individual has overeating behavior in a stressful situation is there is a significant gap between the individual’s actual state and the ideal standards. In order to avoid the negative internal experience brought about by this gap, individuals shrink their attention to the negative stimulus in environment, and this effort to avoid unpleasant emotions by narrowing the cognitive range will weaken the usual inhibitions around food [87–89]. Another possible explanation for the phenomenon of overeating is comfort eating. Comfort eating is eating induced by affect which has been a core theme of explanations for overeating [90]. Previous studies shows that mood improve after food consumption [91]. Therefore, overeating may product positive emotions. Research has demonstrated that both animals and humans tend to eat comfortably nutrient-dense food in response to stress [92]. As such, it makes conceptual sense that coronavirus stress —a sudden stressor [93, 94]—may lead to overeating. This coping activity act to reduce the psychological burden imposed by emotional distress, release cognitive resources to adjust and deal with everyday activities, which have not stopped during the COVID-19 pandemic [95–97]. Based on previous researches, when the level of college students' Coronavirus stress is lower, as their sense of control increase, their levels of overeating tend to decline rapidly; when the level of college students' Coronavirus stress is higher, as their sense of control increase, their levels of overeating tend to decline slower. To date, no previous studies examined whether Coronavirus stress as a moderator in the indirect relation between life history strategy and overeating via sense of control.

### Present study

**Fig. 1:**
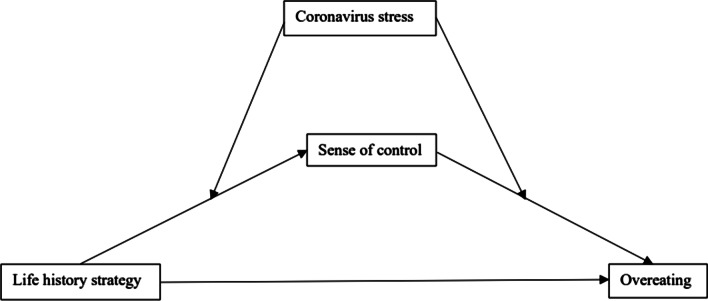
The proposed moderated mediation model

The purposes of this research were twofold: (a) to test whether sense of control would mediate the relation between life history strategy and overeating in college students, and (b) to test whether the indirect relations between life history strategy and overeating via sense of control were moderated by Coronavirus stress. The proposed model is illustrated in Fig. 1. Based on the review of literature, we posit the following hypotheses:

#### **Hypothesis 1**

Sense of control will mediate the relation between life history strategy and overeating.

#### **Hypothesis 2**

Coronavirus stress will moderate the indirect relations between life history strategy and overeating via sense of control.

## Method

### Participants

A total of 3310 college students in China (*M*_*age*_ = 19.74, *SD* = 1.50, range = 16–25, 53.30% Female) participated in this study. For students under the age of 18, we have obtained the consent of the student and the parents. Chinese college students were recruited from more than 30 different universities in 26 regions of China such as Jiangxi, Jilin, Shandong province, etc. The sample source area includes both severely affected areas and mildly affected areas. The sample includes students from “Double First-Class” universities and ordinary universities*.* The samples were collected in February 2020. The sample size needed for a study depends on many factors, including the size of the model, distribution of the variables, amount of missing data, reliability of the variables, and strength of the relations among the variables [98]. According to Kline [99], more complex models, or those with more parameters, require bigger sample sizes than simpler models with fewer parameters. This is because models with more parameters require more estimates, and larger samples are necessary in order to the computer to estimate the additional parameters with reasonable precision. Fritz and MacKinnon [100] provided researchers with sample size requirements for various sizes of the *a* and *b* path in the single mediator model, and they showed that sample size requirements can be very large (i.e., ranged from 462 to 667), especially if small mediated effects are to be detected. For example, when bias-corrected bootstrap is used, the sample size requirement is 462. Considering that the moderated mediation model is more complex than the simple mediation model, we use a larger sample size in the current study. Participation in this study was entirely voluntary and no compensation was given to participants for their participation. To abide by local government policies, the study questionnaire was distributed to potential participants electronically via Survey Star (Changsha Ranxing Science and Technology, Shanghai, China) and no face-to-face contact was made. All participants consented to participation and data were anonymized. Furthermore, 35.40% of these participants were 1st year standing, 26.40% were 2nd year standing, 19.80% were 3rd year standing, and 18.40% were 4th year standing.

### Measures

#### Life history strategy

The Chinese-version [101] of Mini-K scale [49] consists of 20 items (e.g., I have frequent contacts with my friends). The original scale measures the relative position of an individual on a continuum of fast and slow strategies based on 20 psychological and behavioral indicators. Mini-K scale is a part of Arizona life history battery which designed to measure individual’s life history strategy [102]. The reliability and validity of this scale are good [103, 104]. The Chinese version of the Mini-K scale was semantically revised by several fellow teachers and students based on the life history theory [101]. Cronbach’s alpha of the Chinese-vision Mini-K scale were 0.79 [101]. Studies show that Chinese-vision Mini-K scale has good reliability and criterion validity [55, 105, 106]. Cronbach’s alpha of the present Mini-K scale was 0.93. Confirmatory factor analysis (CFA) suggested that the one-factor model fit the data well: RMSEA = 0.07, CFI = 0.96, TLI = 0.93, SRMR = 0.04, which indicated that the validity of this scale was good.

#### Overeating

The overeating was measured by loss of control eating subscale of three-factor eating scale [107]. This scale comprises 9 items (e.g., when I smell a delicious food, I find it very difficult to keep from eating, even if I have just finished a meal.), and all items are limited to the time after the outbreak of the new coronavirus disease. Participants rated each item on a four-point scale ranging from 1 (very inconsistent) to 4 (very consistent) with higher scores showing higher levels of overeating. The scale has good reliability, validity and applicability in the study of Chinese population [41, 108]. In this study, the internal consistency coefficient α = 0.899. Confirmatory factor analysis (CFA) suggested that the one-factor model fit the data well: RMSEA = 0.045, CFI = 0.99, TLI = 0.98, SRMR = 0.01.

#### Sense of control

Sense of control was assessed by the Chinese-version [109] of the Sense of Control Scale [110] which consists of 12 items and includes two dimensions: personal mastery, and perceived constraints (e.g., “I can do almost anything I am determined to do”, “what I can and cannot do is mostly determined by others”). Participants rated each item on a seven-point scale ranging from 1 (very inconsistent) to 7 (very consistent). The scale has good reliability, validity and applicability in the study of Chinese population [55, 111, 112]. In this study, the internal consistency coefficient α = 0.91.

#### Coronavirus stress

The Coronavirus stress was assessed by Coronavirus Stress Measure (CSM) [113]. The scale has 5 items (e.g., how often have you felt that you were unable to control the important things in your life due to the COVID-19 pandemic?). Participants rated each item on a five-point scale ranging from 0 (never) to 4 (always) with higher scores showing higher levels of Coronavirus stress. Confirmatory factor analysis (CFA) suggested that the one-factor model fit the data well: CFI = 0.98, TLI = 0.96, SRMR = 0.07 [113]. The results of the confirmatory factor analysis indicated that the validity of this scale was good [103, 104]. The translation process of the Coronavirus Stress scale was as follows: Two psychology postgraduate with high English proficiency translated the Coronavirus Stress scale into Chinese, and a psychology professor and three psychology PhD compared English Coronavirus Stress scale with Chinese Coronavirus Stress scale, and revised some ambiguities. Two English majors’ postgraduates back-translated and revised the Chinese Coronavirus Stress scale. Researches indicate that the scale is psychometrically adequate and has good reliability [114, 115]. In this study, the Cronbach alpha coefficient was 0.93. Confirmatory factor analysis (CFA) of this study suggested that the one-factor model fit the data well: RMSEA = 0.04, CFI = 0.99, TLI = 0.99, SRMR = 0.004, which indicated that the validity of this scale was good.

#### Procedure

This study was approved by the Research Ethics Committee of the first author’s institution. We obtained consent from all participating college students before data collection. Participants were given the survey questionnaire which they provided demographic information and completed the measurements listed above.

#### Data analysis

The hypothesized moderated mediation model (see Fig. 1) was tested in a single model using a bootstrapping approach to assess the significance of the indirect effects at differing levels of the moderator [116]. Life history strategy was the predictor variable, with sense of control as the mediator. The outcome variable was overeating and coronavirus stress was the proposed moderator. Moderated mediation analyses test the conditional indirect effect of a moderating variable (i.e., coronavirus stress) on the relationship between a predictor (i.e., slow life history strategy vs fast life history strategy) and an outcome variable (i.e., overeating) via potential mediators (i.e., sense of control). The "PROCESS" macro, model 58.v 3.5 [116] in SPSS ver 23 with bias-corrected 95% confidence intervals (*n* = 5000) was used to test the significance of the indirect (i.e., mediated) effects moderated by coronavirus stress, i.e., conditional indirect effects. This model explicitly tests the moderating effect on the predictor to mediator path (i.e., path *a*). An index of moderated mediation was used to test the significance of the moderated mediation, i.e., the difference of the indirect effects across levels of coronavirus stress [116]. Significant effects were supported by the absence of zero within the confidence intervals.

## Results

### Preliminary analyses

The means and Pearson-correlations among the study variables are presented in Table 1. Both life history strategy and sense of control were negatively associated with college students’ Coronavirus stress (*r* = − 0.20, *p* < 0.001; *r* = − 0.19, *p* < 0.001) and positively associated with overeating (*r* = 0.40, *p* < 0.001). Overeating was negatively associated with college students’ life history strategy (*r* = − 0.25, *p* < 0.001) and sense of control (*r* = − 0.24, *p* < 0.001). Sense of control was positively associated with life history strategy (*r* = 0.45, *p* < 0.001).

**Table 1:** Descriptive statistics and correlations among variables (*N* = 3310)

Variables	*M*	*SD*	1. Life history strategy	2. Sense of control	3. Overeating	4. Coronavirus stress
1.Life history strategy	5.42	0.80	–			
2.Sense of control	5.12	0.61	0.45***	–		
3.Overeating	2.07	0.54	− 0.25***	− 0.24***	–	
4.Coronavirus stress	2.00	0.80	− 0.20***	− 0.19***	0.40***	–

*p < .05; **p < .01; ***p < .001

### Testing for mediation effect

Hypothesis 1 assumed that sense of control mediates the relation between life history strategy and overeating. To test this hypothesis, we used Model 4 of the SPSS macro-PROCESS complied by Hayes [116]. The regression results for testing mediation are reported in Table 2. Results indicated that life history strategy were positively related to sense of control. The residual direct effect of life history strategy on overeating remained negative. These results showed that sense of control partially mediated the association between life history strategy and overeating (indirect effect = − 0.0.02, *SE* = 0.003, 95% *CI* = [− 0.03, − 0.017]), and the mediation effect accounted for 29.15% of the total effect of life history strategy on overeating. Therefore, Hypothesis 1 was supported.

**Table 2:** Testing the mediation effect of sense of control on overeating

Predictors	Sense of control		Overeating	
	*B*	*t*	*B*	*t*
Life history strategy	0.20	28.87***	− 0.05	− 9.52***
Sense of control			− 0.11	− 8.74***
*R*^*2*^	0.20		0.08	
*F*	833.32***		151.14***	

*N* = 3310. Each column is a regression model that predicts the criterion at the top of the column

***p < 0.001

### Tests of conditional indirect effects

Hypothesis 2 proposed that the indirect relationships between life history strategy and overeating via sense of control would be moderated by Coronavirus stress. Table 3 showed that the product (interaction term) of life history strategy and Coronavirus stress had a significant negative association with sense of control.

**Table 3:** Testing the moderated mediation effect of LHS on Overeating

Predictors	Sense of control		Overeating	
	*B*	*t*	*B*	*t*
Life history strategy	0.33	17.66***	− 0.04	− 6.93***
Coronavirus stress	1.28	6.67***	− 0.99	− 6.18***
LHS × CS	− 0.01	− 7.79***		
Sense of control			− 0.30	− 10.99***
SC × CS			0.02	8.96***
*R*^*2*^	0.23		0.22	
*F*	323.53***		232.29***	

*N* = 3310. Each column is a regression model that predicts the criterion at the top of the column

*LHS* life history strategy, *SC* sense of control, *CS* Coronavirus stress

**p < 0.01; ***p < 0.001

The hypothesized moderated mediation model was tested using the PROCESS macro mode number 58, which tests a model whereby coronavirus stress moderates the effect of path *a* and *b* (Fig. 1; [116]). The coronavirus stress moderated the indirect relationships between life history strategy and overeating via sense of control. Separately, coronavirus stress moderated the effect between life history strategy and sense of control (Unstandardized interaction *B* = − 0.013, *Bse* = 0.002, *t* = − 7.79, *p* < 0.001, 95% *CI*_boot_ = [− 0.02, − 0.01]), and coronavirus stress moderated the effect between sense of control and overeating (Unstandardized interaction *B* = 0.02, *Bse* = 0.02, *t* = 8.95, *p* < 0.001, 95% *CI*_boot_ = [0.02, 0.03]). Higher sense of control was associated with less overeating (*B* = − 0.30, *Bse* = 0.03, *t* = − 5.31, *p* < 0.001, 95% *CI*_boot_ = [− 0.35, − 0.24]). As zero is not within the Cl, this indicates a significant moderating effect of coronavirus stress on life history strategy on the indirect effect via sense of control [116].

**Fig. 2:**
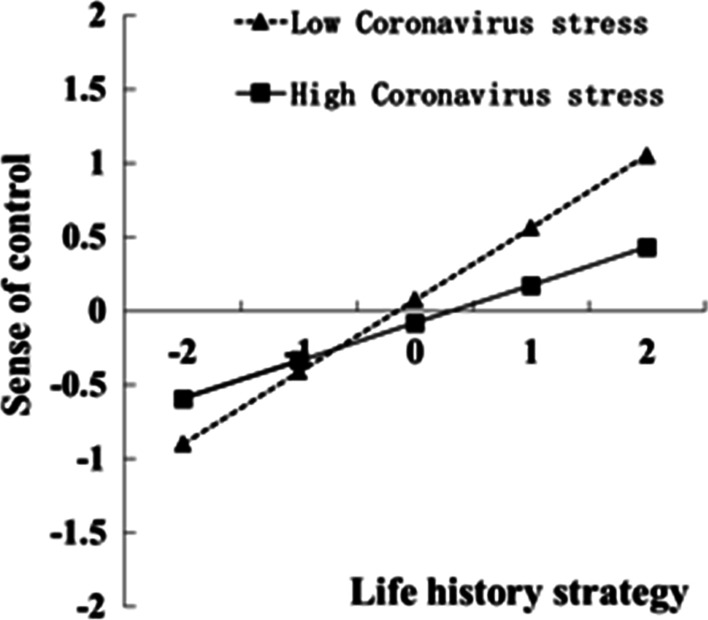
Interaction graphs for indirect paths. *Note*: on the X axis: a positive number = slow life history strategy; a negative number = fast life history strategy; on the Y axis: a positive number = high sense of control; a negative number = low sense of control

**Fig. 3:**
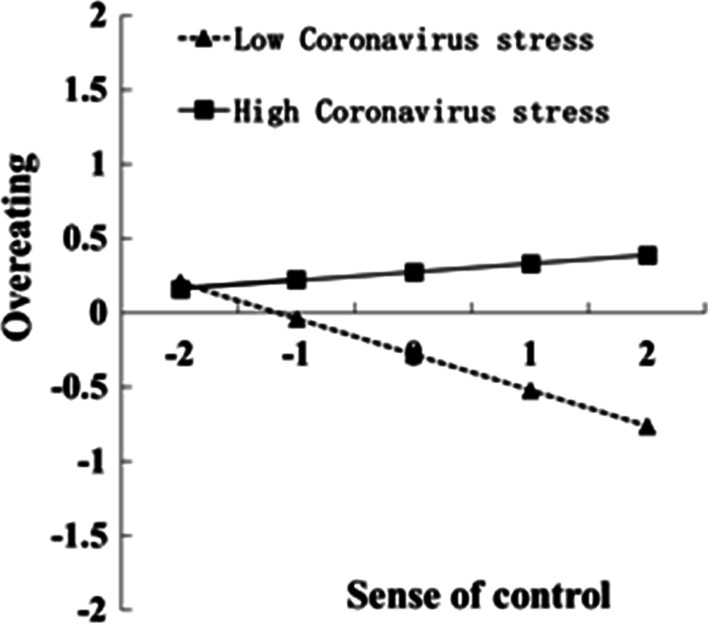
Interaction graphs for indirect paths. *Note*: on the X axis: a positive number = high sense of control; a negative number = low sense of control; on the Y axis: a positive number = high level of overeating; a negative number = low level of overeating

For a clear demonstration of the moderating role of Coronavirus stress, this study plotted the predicted overeating against sense of control, separately for low and high levels of Coronavirus stress (one *SD* below and one *SD* above the mean, respectively; Figs. 2, 3). Simple slope tests revealed that sense of control significantly correlated with overeating (*B* = 0.26, *Bse* = 0.01, *t* = 23.51, 95% *CI*_boot_ = [0.24, 0.28]) for college students with lower level of coronavirus stress. For college students with higher Coronavirus stress levels, life history strategy yielded a weaker association with sense of control (*B* = 0.12, *Bse* = 0.01, *t* = 11.17, 95% *CI*_boot_ = [0.10, 0.15]). The bias-corrected percentile bootstrap analysis further indicated that the indirect effect of life history strategy on overeating through sense of control was moderated by Coronavirus stress. Particularly, for college students low in Coronavirus stress, the indirect effect of life history strategy on overeating via sense of control was significant (*B* = − 0.18, *Bse* = 0.02, 95% *CI*_boot_ = [− 0.21, − 0.15]). The indirect effect for college students with high Coronavirus stress was weaker (*B* = 0.07, *Bse* = 0.05, 95% *CI*_boot_ = [0.011, 0.082]).

## Discussion

While several empirical studies have shown the effect of life history strategy on overeating [41, 44, 45], the underlying mediation and moderation mechanisms remain less clear. Investigating the degree to which these underlying mechanisms of the overeating levels of college students with different life history strategy is vitally important during the COVID-19 period. The present study examined a novel moderated mediation model to test how life history strategy generate harmful overeating, whether this relation was mediated by sense of control, and how Coronavirus stress impact the effects of life history strategy on sense of control and the effects of sense of control on overeating. Our results indicated that slow life history strategy increased sense of control, which in turn, decreased overeating. Coronavirus stress may serve as a risk factor that enhance the fast life history strategy on sense of control during the COVID-19 period.

### The mediating role of sense of control

Mediation results of this study suggested that sense of control was not only an outcome of life history strategy but also a partial catalyst for overeating. For the first stage of the mediation process (i.e., life history strategy → sense of control), this study indicated that slow life history strategy promoted the activation of sense of control mechanisms during the COVID-19 period. This finding is in line with prior literature on sense of control and overeating, whereby sense of control are partly mutable in response to overeating [58–60]. This may be due to individuals with fast life history strategies potentially being more likely to feel that the future is beyond their control. Therefore, they may feel less sense of control over life and their future during the COVID-19 period. Individuals with slow strategy may have more successful life experience and make them believe that they are more likely to decide own future, and thus may have a stronger sense of control over their life [55, 117]. Indeed, those who have fast life history strategy may be more sensitive to the influence of external environment (e. g. COVID-19) which may lead to loss sense of control.

For the second stage of our mediation model (i.e., sense of control → overeating), the present study found that sense of control was associated with overeating. In the context of the COVID-19 crisis, individuals’ sense of control is more difficulty to maintain [78]. According to an experimental study, individuals who loss of sense of control showed a trend towards eating faster, and reported greater desire to eating, feelings of hunger, and liking of higher carbohydrate food intake than control group [118], In other words, the sense of control may influence the individual’s overeating. In summary, individuals with slow life history strategy may be more likely to have long-term goals which may led to higher levels of sense of control and may avoid overeating behaviors, while individuals with fast life history strategy tend to pursue timely satisfaction which may led to lower levels of sense of control and more overeating behaviors during the COVID-19 period. There is a possible explanation for this finding. For people with fast life history strategy, coronavirus stress may make college student`s desire for instant gratification stronger and more difficult to rationally plan for future events, and lower sense of control. Thus, coronavirus stress may act as a risk enhancing fast life history strategy on sense of control among college students. For the second stage of the moderated mediation model pattern were consistent with the charcoal in the snow model [119] and showed that the adverse effects of sense of control on overeating were weaker among college students with high coronavirus stress than in those with low coronavirus stress. Coronavirus stress may serve as a risk factor that enhance the adverse effect of sense of control on overeating. The escape theory is a possible explanation for this finding. For people with lower sense of control, coronavirus stress may make college students to have more difficulty to control themself which may led to more overeating behaviors. Thus, coronavirus stress act as a risk increasing sense of control on overeating among college students during COVID-19 period.

It is also worth noting, however, that sense of control only partially mediated the association between life history strategy and overeating. That is, life history strategy remained a significant, direct predictor of overeating even upon controlling for sense of control. The remaining direct and negative association between life history strategy and overeating may suggest that slow life history strategy are uniquely salient factors that could significantly decrease the prevalence of overeating during the COVID-19 period. Thus, each of the separate paths in the mediation model was noteworthy and practical implications may be necessary at various stages of managing one’s coronavirus stress to mitigate susceptibility to overeat. The following are the specific practical implications. The society can guide college students to adopt acceptance strategies to relieve coronavirus stress. The acceptance strategy is about accepting the situation (recognizing the reality of the stressor) and learning how to live in it without actively trying to change the situation [120]. In addition, relevant department can build an online psychological consultation platform through new media technology to improve the individual's ability to cope with stress [105, 121].

### The moderating role of coronavirus stress

A study in the United States shows that individuals who experience high levels of COVID-19 related pressures believe that their eating habits are worse than before the COVID-19 pandemic [122]. Our findings suggested that coronavirus stress could moderate the relation between life history strategy and sense of control as well as the path between sense of control and overeating. For the first stage of the moderated mediation model pattern were consistent with the Drop in the bucket model which means that the moderating variable could increase the adverse effect of the independent variable on the moderate variable [119]. Our results showed that the effect of fast life history strategy on sense of control were weaker among college students with high coronavirus stress than those with low coronavirus stress. Coronavirus stress may serve as a risk factor that decrease slow life history strategy on sense of control during the COVID-19 period. There is a possible explanation for this finding. For people with slow life history strategy, coronavirus stress may make college student`s desire for instant gratification stronger and more difficult to rationally plan for future events, and lower sense of control. Thus, coronavirus stress may act as a risk decrease slow life history strategy on sense of control among college students. For the second stage of the moderated mediation model pattern were consistent with the charcoal in the snow model [119] and showed that the adverse effects of sense of control on overeating were weaker among college students with high coronavirus stress than in those with low coronavirus stress. Stress acts as a moderator, reducing the protective effect of high sense of control against overeating, i.e., as stress increases, the protective effect of high sense of control is diminished and overeating increases. The escape theory is a possible explanation for this finding. For people with lower sense of control, coronavirus stress may make college students to have more difficulty to control themself which may led to more overeating behaviors. Thus, coronavirus stress acting as a risk increasing sense of control on overeating among college students during COVID-19 period.

Based on the life history theory, this study reveals the influence of psychological instinct on individual behaviors, and provides an evolutionary explanation for this path. The findings have theoretical and practical contribution. In the context of the COVID-19 pandemic, the study deepens the theory’s application and interpretation in the field of eating behaviors in the Chinese context. By exploring the role of the cognitive tendency of life history strategies, we can provide evidence for the intervention of cognitive strategies for overeating; it can also reduce the possibility of overeating by enhancing the individual's sense of control; reducing the individual's coronavirus stress can also reduce the possibility of overeating.

### Limitations and future directions

Interpretation of the findings of this study must consider the following limitations. Firstly, the fact that this study is a cross-sectional survey study does not allow inference of causality. Future studies may examine longitudinal data as they become available to infer causality of the findings in this study. Secondly, the measures are based on a Chinese college student sample and the extent to which results may be generalizable to other cultural contexts or demographics is limited. Future studies may collect data from multiple informants (e.g., teacher, peer, or parent) across different cultures to further examine the robustness of our findings. Additionally, the current study did not collect information regarding students’ majors nor family socioeconomic status. Prior studies have identified both healthcare related majors and family socioeconomic status to be relevant factors in COVID-19-related stress and behaviors [123, 124] and future studies may incorporate this. Future studies may also examine the robustness of the model presented in this paper in other populations that may be exposed to qualitatively different COVID-19 stressors in both frequency and intensity (e.g., frontline healthcare workers), and longitudinal studies of eating behaviour and other behavioural risk factors are needed to understand both short-term and long-term mental and physical health impacts. In addition, future research should measure the individual’s eating disorder level to make the research results more accurate.

## Conclusion

Although further replication and extensions are needed, this study is an important step in unpacking how life history strategy relate to overeating in Chinese college students. Because sense of control served as one potential mechanism by which life history strategy were associated with overeating, it remains important to directly or indirectly reduce coronavirus stress to enhance the impact of sense of control on overeating. Accordingly, intervention programs should aim to help individuals develop resilience or effective coping mechanisms against COVID-19 related stressors to promote mental wellbeing. Psychological assistance under the COVID-19 pandemic should start from reducing the individual's coronavirus stress, such as using cognitive behavioral therapy [125], etc.

## Data Availability

This study data is available to researchers.

## Abbreviations

LHT: Life history theory
LHS: Life history strategy
SC: Sense of control
CS: Coronavirus stress
